# Prevalence and associated factors of low birth weight in Axum town, Tigray, North Ethiopia

**DOI:** 10.1186/s13104-018-3801-z

**Published:** 2018-10-01

**Authors:** Wondim Aboye, Tesfay Berhe, Taddis Birhane, Hadgu Gerensea

**Affiliations:** 1 School of Public Health, College of Health Science, Aksum University, Aksum, Ethiopia; 2 School of Nursing, College of Health Science, Aksum University, Aksum, Ethiopia

**Keywords:** LBW, Aksum, Ethiopia

## Abstract

**Objective:**

Weight at birth is a good indicator of the newborn’s chances for survival, growth, long-term health and psychosocial development. Therefore, the aimed of this study is to assess the prevalence and associated factors of low birth weight in Axum town, Tigray, North Ethiopia.

**Result:**

The magnitude of low birth weight was 8.8%. Height of mother adjusted odds ratio (AOR) 4.607 (CI 1.34–15.8), gestational age AOR 4.7 (CI 1.08–20.44), anti-natal care (ANC) visit AOR 0.076 (CI 0.009–0.645), anemia during pregnancy AOR 14.5 (CI 3.821–55.6) and drinking alcohol AOR 6.4 (CI 1.235–33.94) were found to be significantly associated with low birth weight. Pre-conceptual counseling on nutrition, about the effect of short suture on birth outcome and personal maternal habit (drinking alcohol), effective treatment and prevention of anemia and awareness on the importance ANC follow up should be the target.

**Electronic supplementary material:**

The online version of this article (10.1186/s13104-018-3801-z) contains supplementary material, which is available to authorized users.

## Introduction

Low birth weight (LBW) is defined by the World Health Organization as weight at birth < 2500 g (5.5 lb). There is significant fluctuation in the number of low birth weight across regions and within state; however, the great figure of low birth weight births happen in low- and middle-income countries and particularly in the most vulnerable populations [1].

Based on national and regional estimates in Ethiopia 2,600,000 live births are reported out of these live births 530,400 infants are LBW with mortality rate of 32.4%. In Ethiopia the low birth weight (LBW) estimate has risen from 2000 to 2005 from 15.0 to 20.3% with 1.1% increase per year [2]. In Ethiopia, having one of the highest neonatal mortality rate in the world, recent estimates shows that the median prevalence of low birth weight is around 11% and ranges high up as 23%. Mother’s height, mother’s weight, anemia, physical work, tobacco chewing, and history of abortion are significant determinants of LBW [3].

This study will seek to ascertain the prevalence of low birth weight, describe the socio-demographic characteristics of women attending delivery and to investigate the factors contributing to LBW in Aksum town. The findings will create awareness in the community about the problem and contribute towards formulating locally appropriate interventions to prevent low birth weight. The findings will be shared among various stakeholders to stimulate focused intervention programs.

## Main text

### Methodology

#### Study area and design

Institutional based cross-sectional study was conducted in St. Marry hospital and Aksum University specialized and comprehensive hospital, Aksum, Tigray region regional state, Ethiopia, Which is the capital town of central zone of Tigray regional state. Aksum is found at a distance of 1024 km from Addis Ababa, capital city of Ethiopia and 241 km north of Mekelle, the capital city of Tigray [4–14].

#### Study period

The study was conducted from April1/2018 to May30/2018.

#### Source population and study population

All mothers who give birth at Aksum St. Marry hospital and Aksum university comprehensive specialized hospital, central zone of Tigray and those who randomly selected mothers were the study participant.

#### Sample size determination

A total 308 samples were calculated using the formula to estimate a single Population proportion.

With the assumption of 95% confidence interval (CI), d = 0.035 and prevalence of LBW of the new born in Aksum town research shows 9.9%.

#### Sampling procedure and technique

The calculated sample size (308) was proportionally allocated to these health facilities based on delivery case load of each health facility. Finally, data was collected systematically by skipping every third neonate until the needed sample size was found.

#### Data collection procedures

The data was collected using structured, pretested interviewer administered questionnaire containing socio-demographic characteristics and other variables associated with delivering LBW baby. Data was collected through interviewing the mother and measuring the weight of the newborn, height, weight and mid-upper arm circumference (MUAC) of the mother (see Additional file 1).

##### Dependent variable

Low birth weight.

##### Independent variable

- Demographic and socio-economics: characteristics, including newborn sex, maternal age, family monthly income, educational level, maternal occupation, religion, marital status ethnicity.
- Maternal anthropometry (nutritional status): pre-pregnancy weight, maternal MUAC, maternal height, maternal weight.
- Obstetric and gynecologic factor: parity, birth interval, history of abortion, anemia, chronic illness of the mother, gestational age at birth, no of anti-natal care (ANC) follows up.
- Maternal behavior: khat, alcohol, physical activity.

#### Data management, processing and analysis

The data was entered to Statistical Package for Social Sciences (SPSS) version 21.0. Identification of implausible values and missing data or possibly miss-coded data was cross-checked. After cleaning the data, frequencies and percentages was calculated to all variables. Bivariate analysis; cross tabulations was done to see the association between the explanatory and outcome variables. Multivariable logistic regression was employed by selecting only variables that appeared to be statistically significant at (P < 0.05) in the bivariate analysis. Model fit was checked by Hosmer–Lemeshow goodness-of-fit test.

Finally, the data was presented with appropriate tables, diagrams and figures. Adjusted odd ratio (AOR) was calculated to check strength of association and P < 0.05 was taken as significant association.

### Results

#### Socio demographic characteristics of the respondents

Majority of mothers 208 (67.5%) were aged 20–34. All of the mothers were Tigrean in ethnic. Majority of the mothers were married 286 (92.9). Out of the respondent 298 (96.1%) were Orthodox Christians. Majority of the respondent 157 (51%) of mothers could not read and write and 133 (43.3%) of mothers were housewife. More than half of mothers 188 (61%) respondents were residing in urban areas. Based on the findings from the income distribution higher percentage of mothers 95 (30%) had an average household income of < 1000 ETB per month (Table 1).

**Table 1:** Distribution of mothers by socio demographic characteristics in Axum St. Marries hospital and Aksum university comprehensive specialized hospital, Tigray, Northern Ethiopia, 2018

Variables	Categories	Frequency	Percentage (%)
Age of mothers (years)	< 20	80	26
	20–34	208	67.5
	≥ 35	20	6.5
Religion	Orthodox Christian	296	96.1
	Muslim	9	2.9
	Others	3	1.0
Educational status	Unable to read and write	157	51
	Able to read and write	17	5.5
	Primary school (1–8)	34	11
	Secondary school (9–12)	61	19.8
	College and above	38	12.3
Marital status	Single	5	1.6
	Married	286	92.9
	Divorced	3	1.0
	Separated	9	2.6
	Widowed	5	1.6
Occupation	Employed	67	21.8
	Housewife	133	43.2
	Farmer	91	29.5
	Merchants	14	4.5
	Daily laborer	3	1
Monthly income (Birr)	< 500	52	16.9
	500–1000	95	30.8
	> 1000–2000	78	25.3
	> 2000	83	26.9
Residence	Urban	188	61
	Rural	120	39

#### Bivariable and multivariable logistic regression analysis of factors associated with LBW

In bivariate analysis, religion, residence, height of mother, weight of mother, gestational age, ANC visit, anemia during pregnancy, drinking alcohol and frequency of alcohol drinking were found to be associated with low birth weight.

But, in multivariate analysis, only height of mother AOR 4.607 (CI 1.34–15.8), gestational age AOR 4.7 (CI 1.08–20.44), ANC visit AOR 0.076 (CI 0.009–0.645), anemia during pregnancy AOR 14.5 (CI 3.821–55.6) and drinking alcohol AOR 6.4 (CI 1.235–33.94) were found to be significantly associated with low birth weight.

Mothers whose height < 150 cm was 4.6 times more likely to deliver low birth weight baby than mothers with height of ≥ 150 cm (AOR 4.607 (CI 1.34–15.8)). The chance of LBW delivery were 4.7 times higher for neonate with gestational age < 37 weeks 4.7 (CI 1.08–20.44). Mother who had ANC follow up had 92.4% less chance to have low birth weight baby AOR 0.076 (CI 0.009–0.645) than those who didn’t have. Mothers who had anemia during current pregnancy were 14 times more likely to deliver low birth weight neonate compared to mothers without anemia AOR: AOR 14.5 (CI 3.821–55.6). Mothers who drink alcohol during current pregnancy were 6.4 times more likely to deliver low birth weight neonate compared to mothers who didn’t drink AOR 6.4 (CI 1.235–33.94). Further see Table 2.

**Table 2:** Bivariable and multivariable logistic regression analysis of factors associated with LBW in Axum St. Marry Hospital and Aksum university comprehensive specialized hospital northern Ethiopia, 2018

Variables	Characteristics	Birth weight		COR	P-value	AOR	P value
		NBW	LBW				
		FR (%)	FR (%)				
Religion	Orthodox	271 (91.6%)	25 (8.4%)	21.7046 (0.004–0.527)	0.013	0.195 (0.009–4.092)	0.293
	Others	10 (83.3%)	2 (16.7%)	2.000 (–)	1	0.000 (000–0.0001)	1
Residence	Urban	177 (94.1%)	11 (5.9%)	0.4 (0.181–0.903)	0.027	1.100 (0.333–3.636)	0.876
	Rural	104 (86.7%)	16 (13.3%)	0.154 (–)	1	–	–
Height of mother (cm)	< 150	34 (68%)	16 (32%)	10.5 (4.529–24.655)	0.00	4.607 (1.34–15.8)*	0.015
	≥ 150	247 (95.7%)	11 (4.3%)	0.045 (–)	1	–	1
Weight of mother (kg)	< 50	97 (86.6%)	15 (13.4%)	2.37 (1.068–5.266)	0.034	0.614 (0.173–2.187)	0.452
	≥ 50	184 (93.9%)	12 (6.1%)	0.065 (–)	1	–	1
GA (weeks)	< 37	17 (63%)	10 (37%)	9.135 (3.632–22.975)	0.0	4.7 (1.08–20.44)*	0.038
	≥ 37	264 (94%)	17 (6%)	0.064 (–)	1	–	1
ANC visit (visit)	≥ 4	225 (93.8%)	15 (6.2%)	0.1 (0.016–0.645)	0.015	0.076 (0.009–0.645)*	0.018
	< 4	55 (83.8%)	12 (16.2%)	0.938 (0.133–6.628)	1	0.923 (0.090–9.479)	1
Anemia during px	Yes	17 (58.6%)	12 (41.4%)	12.3 (5.013–30.556)	0.0	14.5 (3.821–55.6)*	0.0
	No	264 (96%)	14 (4%)	(–)	1	–	1
Drinking alcohol	Yes	41 (82%)	9 (18%)	2.9 (1.231–6.958)	0.015	6.4 (1.235–33.94)*	0.027
	No	240 (93%)	18 (7%)	0.000 (–)	1	–	1
Amount of alcohol	3 times per week	240 (93%)	18 (7%)	0.18 (0.057–0.567)	0.03	0.274 (0.016–4.617)	0.369
	Once a month	29 (87.8%)	4 (12.2%)	0.369 (0.060–2.274)	1	0.274 (0.016–4.617)	0.369

1 = reference category, others  = Muslim, protestant

* Significantly associated with P < 0.05

### Discussion

Low birth weight observed in this study was consistent with studies conducted in Iran (8.8%), Jimma (11.02%), [15] Nigeria (7.3%), [16], Laelay Maichew (6.6%) and Axum district (9.9%) [17]. LBW EDHS report of Ethiopia in 2011 shows 11%. The possible explanation between the variations might be the difference in geographical variation which might had difference in health service utilization and nutritional status of mothers during pregnancy.

But it had relatively lower when compared with study finding done in India, Uttar Pradesh which was about 40% [18] and study conducted in Kersa demographic and Surveillance and Health research Center (KDS–HRC) which showed an incidence of 28.3% [19]. The possible explanation for the decrement in LBW in the current study might be the study done in Kersa was done in rural area. The other explanation might be due to variation in characteristics of study population like nutritional status and maternal feeding habit.

In this study, anemia during pregnancy, height of the mother < 150 cm, gestational age at birth < 37 weeks, number of ANC visit and drinking alcohol were found to be significantly associated with low birth weight.

Mothers who had anemia during current pregnancy were 14 times more likely to deliver low birth weight neonate compared to mothers without anemia (AOR: 14.5 (CI 3.821–55.6). This finding consistent with study conducted in Deberbirhan Ethiopia and Sudan [20, 21]. Anemia also found to increase chance of LBW in which the chance of LBW among pregnant women with anemia was seen in Nigeria [19]. This might be because of that micronutrient deficiencies during pregnancy had been shown to have serious implications on the developing fetus. So mothers with anemia were more likely to had LBW baby.

Gestational age plays an important role in determining infants ‘birth weight. Infants who are delivered prematurely (< 37 weeks) are at higher risk to have low birth weight infants. The World Health Organization estimated about one-third of low birth weight infants is caused by prematurity. The results of the present study revealed that the chance of LBW delivery were 4.7 times higher for neonate with gestational age < 37 weeks AOR 4.7 CI 1.08–20.44). This finding is consistent with study conducted in Jimma [15]. This finding is also consistent with the research reported from study done in Italy, Iran and Tanzania, which shows significant association of gestational age with the weight of the newborn [21, 22]. This might be due to did not reach the optimal time which ≥ 37 weeks of gestation and the body weight of the fetus falls due to prematurity.

One of the other factors that were found to predispose mothers to have LBW is maternal height. In this study mothers whose height < 150 cm was 4.6 times more likely to deliver low birth weight baby than mothers with height of ≥ 150 cm (AOR 4.607 CI 1.34–15.8). The reported value is consistent with the study finding in Southwestern Ethiopia and Tanzania [5, 19]. Also the study conducted in India which revealed that low birth weights were significantly higher among mothers with height < 150 cm [23]. This can be expected because the shorter the mother may less able to carry full term and this can lead to preterm labor and prematurity. This study also shows that mother who had > 4 times ANC follow up had 92.4% less chance to have low birth weight baby AOR 0.076 (CI 0.009–0.645) than those who have < 4 times ANC follow. By having ANC follow up, other risk factors for LBW also be addressed like anemia, iron supplementation, counseling on danger sign of pregnancy etc. Mothers who drink alcohol during current pregnancy were 6.4 times more likely to deliver low birth weight neonate compared to mothers who didn’t drink AOR 6.4 (CI 1.235–33.94).

### Conclusion

The magnitude of low birth weight in this study was relatively lower than other studies. Anemia during pregnancy, height of the mother < 150 cm, gestational age at birth < 37 weeks, number of ANC visit and drinking alcohol were found to be significantly associated with low birth weight.

#### Limitation of the study

Since this is cross-sectional study it does not show the direction of relationship. Similarly it is sensitive topic and there may be under report.

## Additional file

**Additional file 1.** Questionnaire.

## Abbreviations

LBW: low birth weight
CI: confidence interval
AOR: adjusted odd ratio
SPSS: Statistical Package for Social Sciences
IRB: Institutional Review Board
