# Supplementary material for: Prevalence and associated factors of low birth weight in Axum town, Tigray, North Ethiopia
Source: BMC Res Notes. 2018 Oct 1;11:684. doi: 10.1186/s13104-018-3801-z (PMC6167810; doi:10.1186/s13104-018-3801-z)
Supplement: Supplementary file 1 — Additional file 1. Questionnaire. [file 13104_2018_3801_MOESM1_ESM.docx]

**QUESTIONER** (English Version)

Questionnaire on assessment of the magnitude of low birth weight and associated factors in Axum St. Marry hospital.

1. Questionnaire identification no. _______

2. Address of the client: ______________

INTRODUCTION: How are you? My name is ____. I would like to inform you that you and I would have a short discussion concerning this study. Before we go to our discussion, I will request you to listen carefully to what I am going to read to you about the purpose and general condition of the study and tell me whether you agree or disagree to participate in this study.

Consent form; The purpose of this study is to assess the magnitude and associated factors of low birth weight in Axum St.Marry hospital .The study will be conducted through interviews. I am asking you for a little of your time, about 25 - 30 minutes, to help us in this study. In the end, it is hoped that the information you give us could help to assess the magnitude and associated factors of low birth weight. I would like to assure you that this privacy should strictly be maintained throughout. A code number will identify every participant and no name will be used. Your responses to any of the questions will not be given to anyone else and no reports of the study will ever identify you. If a report of results is published, only information about the total group will appear. The interview is voluntary.

Are you willing to participate in this study?

1. Yes. 2. No

Thank you!!

If the study subject agrees to participate in the study, start the interview.

3. Interviewer signature certifying that informed consent has been given verbally by the respondent.

Name _________________ Signature _____________Date ______________.

Now I would like to begin by asking you a few questions about yourself and your family (answer by encircling)

23

**Checklist for data collection on magnitude and maternal risk factors associated with low birth weight Neonates in Axum St. Marry hospital**

Note the inclusion criteria from clinical records:

▪Is the child born single 1: YES 2: NO

▪Is the child free of any visible major birth defect 1: YES 2: NO

If ―No to any of the inclusion criteria, stop the collection of data.

| No . | Question | Answer | Skip |
| --- | --- | --- | --- |
| 1 | How old are you? | 1. <20 2. 20-34 3. 35+ |  |
| 2 | What is your marital status? | 1. Single 2. Married 3. Divorced 4. Separated 5. Widowed |  |
| 3 | What is your religion? | 1. Orthodox 2. Muslim 3. Others (protestant, catholic) |  |
| 4 | What is the highest level of school you have completed? | 1. Unable to read & write 2. Able to read and write 3. Primary school (1-8) 4. Secondary school (9-12) 5. College and above |  |
| 5 | What is your occupation? | 1. Employed 2. House wife 3. Farmer 4. Merchant 5. Daily laborer |  |
| 6 | What is your residence? | 1. Urban 2. Rural |  |
| 7 | what is your ethnicity | 1. Amhara 2. Tigray 3. Oromo 4. Others |  |
| 8 | How much is your monthly Income | 1. <500 2. 500-1000 3. 1000-2000 4. >2000 |  |
| 9 | What is your height? | 1. <150 cms 2. >=150 cms |  |
| 10 | What was your weight in kg? (recent one) | 1. <50 2. >50 3. Not measured |  |
| 11 | What is birth weight of the your baby in grams? | 1. > = 2500 gms 2. <2500 gms |  |
| 12 | What was the gestational age at delivery, in weeks? Please Calculate from the last menstrual period of the mother or record from ultrasound reading if available. | 1. <37 wks 2. >=37 wks |  |
| 13 | What is your parity? is there any delivery that passed 28 weeks of gestation.  (If she was Gravid 1 skip to 12) | 1. 1 2. 2-3 3. >3 |  |
| 14 | Do you have history of abortions? | 1. Yes 2. no |  |
| 15 | How many times did you receive antenatal care during your current  Pregnancy? | 1. 1 2. 2-3 3. >=4 4. No ANC visiting |  |
| 16 | Do you have any complications occurred during your pregnancy in the list below?   1. Vaginal bleeding 2. Vaginal gush of fluid 3. Severe head ache 4. Blurred vision 5. Fever 6. Abdominal pain   Other (specify): | 1. Yes 2. No |  |
| 17 | During the current pregnancy, for how many times did you take iron tablets? | 1. <3 months 2. >=3 3. Do not take iron |  |
| 18 | During your current pregnancy, have you been told that you have anemia? | 1. Yes 2. No 3. Do not remember |  |
| 19 | Have you ever been told that you have chronic hypertension? | 1. Yes 2. No 3. Do not remember |  |
| 20 | During this pregnancy, have you been told that you have developed pregnancy induced hypertension? | 1. Yes 2. No 3. Do not remember |  |
| 21 | Have you ever been told that you have Diabetes Mellitus? | 1. Yes 2. No 3. Do not remember |  |
| 22 | During your current pregnancy, have you been told that you have developed gestational diabetes mellitus? | 1. Yes 2. No 3. Do not remember |  |
| 23 | What is the sex of the newborn? | 1. Male 2. Female |  |
| 24 | what is your inter Pregnancy interval in years | 1. <2 2. >=2 |  |
| 25 | During your pregnancy, did you take drinks containing alcohol? | 1. Yes 2. No 3. Do not remember |  |
| 26 | How often were you taking alcohol drinks? | 1. Daily 2. 3 times per week 3. 1 times per week 4. Once a month |  |
| 27 | During your current pregnancy, did you ever chew Khat? | 1. Yes 2. No 3. Do not remember |  |
| 28 | How often were you chewing khat? | 1. Daily 2. 3 times per week 3. 1 times per week 4. Once a month |  |
| 29 | During your current pregnancy, did you ever smoke? | 1. Yes 2. No 3. Do not remember |  |
| 30 | How often were you smoking? | 1. Daily 2. 3 times per week 3. 1 times per week 4. Once a month |  |
| 31 | Did you wash your hand before and after preparation of food? | 1.yes  2.no |  |
| 32 | Did you wash your hand before and after visiting toilet? | 1.yes  2.no |  |
| 33 | During your current pregnancy, what type food you used to it? | 1.animal product  2.vegitables |  |
| 34 | During your current pregnancy, how many times you used to ate per day? | - 1. One time   2. Two times   3. Three times   4. >three times |  |
